# Passage of Magnetic Tat-Conjugated Fe_3_O_4_@SiO_2_ Nanoparticles Across In Vitro Blood-Brain Barrier

**DOI:** 10.1186/s11671-016-1676-2

**Published:** 2016-10-10

**Authors:** Xueqin Zhao, Ting Shang, Xiaodan Zhang, Ting Ye, Dajin Wang, Lei Rei

**Affiliations:** 1 College of Life Sciences, Zhejiang Sci-Tech University, Hangzhou, 310018 People’s Republic of China; 2 Department of Biomaterials, College of Materials, Xiamen University, Xiamen, 361005 People’s Republic of China

**Keywords:** Blood-brain barrier, Magnetic nanoparticles, hCMEC/D3 cell, Magnetic targeting, Tat peptide

## Abstract

Delivery of diagnostic or therapeutic agents across the blood-brain barrier (BBB) remains a major challenge of brain disease treatment. Magnetic nanoparticles are actively being developed as drug carriers due to magnetic targeting and subsequently reduced off-target effects. In this paper, we developed a magnetic SiO_2_@Fe_3_O_4_ nanoparticle-based carrier bound to cell-penetrating peptide Tat (SiO_2_@Fe_3_O_4_^﻿-Tat﻿^) and studied its fates in accessing BBB. SiO_2_@Fe_3_O_4_-Tat nanoparticles (NPs) exhibited suitable magnetism and good biocompatibility. NPs adding to the apical chamber of in vitro BBB model were found in the U251 glioma cells co-cultured at the bottom of the Transwell, indicating that particles passed through the barrier and taken up by glioma cells. Moreover, the synergistic effects of Tat and magnetic field could promote the efficient cellular internalization and the permeability across the barrier. Besides, functionalization with Tat peptide allowed particles to locate into the nucleus of U251 cells than the non-conjugated NPs. These results suggest that SiO_2_@Fe_3_O_4_-Tat NPs could penetrate the BBB through the transcytosis of brain endothelial cells and magnetically mediated dragging. Therefore, SiO_2_@Fe_3_O_4_-Tat NPs could be exploited as a potential drug delivery system for chemotherapy and gene therapy of brain disease.

## Background

The blood-brain barrier (BBB) comprised of brain capillary endothelial cells is the most restrictive barrier in vivo that hampers the transport of s ubstances from the peripheral circulation to the brain and helps maintain brain homeostasis [1]. However, essentially 98 % of small-molecule drugs and 100 % of large-molecule drugs do not pass through the physiologic barrier that inhibit drug delivery from blood circulation to brain tissue [2, 3]. Therefore, the development of a novel drug delivery system to aid drugs across the BBB is the crucial point of the treatment of many brain diseases.

Currently, different strategies for bypassing the BBB have been developed, including direct injection into the brain, transient disruption of the BBB, inhibition of efflux pumps, pro-drug strategy, and receptor-mediated transcytosis [4–8]. Nanoparticle-based delivery system has been proved an advantage in effective transportation of various drugs across BBB [9–11], especially iron oxide nanoparticles (IONPs) have attracted significant importance in the last decade due to their intrinsic magnetophoretic mobility, which enable targeting the lesions by magnetic guidance and reducing off-target effects [12]. Because of high surface-to-volume and the magnetic interaction, bare IONPs tended to aggregate and lower magnetic responds by oxidation, which induce a limited drug targeting [13]. Several biocompatible polymers, such as PEG, chitosan, alginate, dextran, and fetal calf serum, have been used to stabilize Fe_3_O_4_ particles and further functionalize [14–16]. Among all the possible surface modifications for IONPs, silica (SiO_2_) coating was supposed especially suitable to be employed for medical purposes due to high pore volume, good biocompatibility, and its transparency [17, 18]. It has been proved that silica coating on IONPs improves cellular uptake and achieves targeted delivery of drug under magnetic field condition [19, 20]. However, low efficiency of permeability and accumulation in the brain is still a great issue.

Cationic cell-penetrating peptides (CPPs) can facilitate the internalization of attached macromolecules and even nano-carriers (e.g., polymers, liposomes) by various cells via independent transporters and receptor-mediated endocytosis [21, 22]. A number of CPPs including trans-activating transcriptional activator (TAT), angiopep, penetratin, rabies virus glycoprotein (RVG), prion peptide, and SynB have already been demonstrated the ability of improving drug delivery across the BBB [23, 24]. Peptide Tat (YGRKKRRQRRR), derived from TAT protein, can increase the permeability of brain endothelial cells by inhibiting occludin expression and cleaving occludin via matrix metalloproteinase-9 [25]. It has been demonstrated that Tat-conjugated nanoparticles can deliver siRNA and drug across the BBB to kill intracerebral malignant glioma cells and further extend the mouse life span [26]. On the other hand, the permeation mechanism and the precise location of the particles across the BBB remain poorly understood at a cellular level [27]. In vitro BBB model may keep the status of BBB integrity, which helps to be understood in terms of how BBB facilitate or interfere with drug delivery [28].

**Scheme 1:**
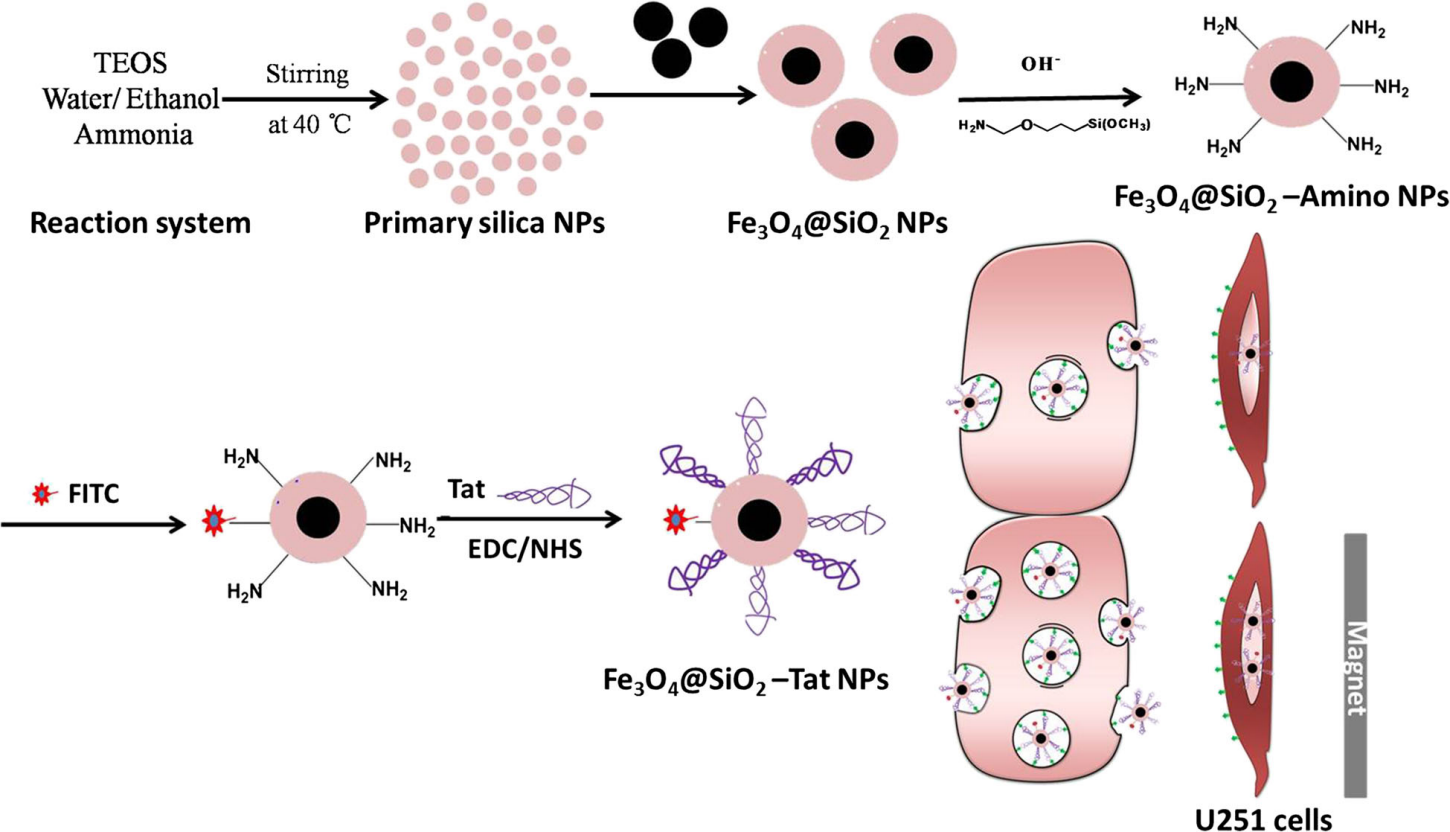
Design and synthesis of Fe_3_O_4_@SiO_2_-Tat magnetic NPs to enhance the permeability across the BBB

In the present study, we developed a novel core-shell structured magnetic Tat-conjugated SiO_2_@Fe_3_O_4_ nanoparticles (NPs). This study aimed to evaluate the nanoparticles’ permeability across BBB and their fates in accessing BBB as shown in Scheme 1. Human brain endothelial cell (hCMEC) monolayer and hCMEC/U251 co-culture model were used to examine cellular uptake, BBB permeability, and subsequently location in U251 cells in the absence and presence of a magnetic field. Besides, the physicochemical properties, cytotoxicity, and the integrity of monolayer were also investigated. The studies of systematic effect of cell-penetrating peptide and magnetic field on mediating BBB permeability and internalization into brain endothelial cells may aid to design peptide-functionalized magnetic NPs for brain targeting in the future.

## Methods

### Materials and Cell Lines

Tetraethyl orthosilicate (TEOS), ferrous chloride (FeCl_3_), ferric chloride (FeCl_2_), and sodium citrate were purchased from Xilong Chemical Co., Ltd. (Shantou, China). 3-Aminopropyl-trimethoxysilane (APTMS) was purchased from Acros Organics (Belgium, USA).

*N*-Hydroxy sulfo-succinimide (NHS), 1-(3-(dimethylamino)-propyl)-3-ethylcarbodiimide hydrochloride (EDC), and FITC-labeled Tat (Tyr-Gly-Arg-Lys-Lys-Arg-Arg-Gln-Arg-Arg-Arg) peptide were provided by Gier Biochemistry Co., Ltd. (Shanghai, China). BCA protein assay kit was purchased from Applygen Technologies Inc. (Beijing, China). External magnetic field was applied by placing an Nd–Fe–B magnet under the culture plates using an immobilized tube apparatus. Human glioma cell lines U251 and human brain capillary endothelial cells hCMEC/D3 (abbreviated as hCMEC) were purchased from BeNa Culture Collection (Beijing, China). All the cells were cultured in RPMI-1640 media with 10 % fetal bovine serum, 10 % penicillin, and 10 % streptomycin at 37 °C in CO_2_ incubator.

### Synthesis of Tat-SiO_2_@Fe_3_O_4_ NPs

The magnetic Tat-SiO2@Fe_3_O_4_ NPs were synthesized as the following steps. The superparamagnetic Fe_3_O_4_ NPs were synthesized firstly by alkaline co-precipitation [29]. Under vigorous stirring at 80 °C, ammonia water was dropped into the mixture solution of Fe(II) and Fe(III) (molar ratio = 2:1) till the pH 10. After 20 min of continued stirring, sodium citrate (Na_3_Cit) was added into the mixture at 40 °C with stirring for 90 min under N_2_ protection to obtain black magnetite Fe_3_O_4_ NPs. Secondly, SiO_2_-coated Fe_3_O_4_ NPs (SiO_2_@Fe_3_O_4_ NPs) were prepared by the Stöber method [30]. The as-prepared 10 mg of Fe_3_O_4_ NPs was ultrasonically dispersed into a mixture of ethanol/water solution (50.7 mL; *v*:*v*, 50:0.7), TEOS(0.3 mL), and NH_4_OH (1.7 mL) and then continuously stirred for 3 h. The obtained NPs were collected using an external magnet and washed sequentially with water and ethanol. Thirdly, the 10 mg SiO_2_@Fe_3_O_4_ NPs was re-dispersed into 10 mL ethanol, and 500 μL of APTMS was added and stirred for 24 h under N_2_ protection at room temperature to introduce amino-silane coating of SiO_2_@Fe_3_O_4_ NPs. Finally, EDC-NHS coupling was used for the attachment of the Tat peptide to amino-functionalized NPs. The as-prepared 100 μL of amino-SiO_2_@Fe_3_O_4_ NPs (1 mg/mL) was activated using EDC (4 nmol) and NHS (4 nmol). Then, 10 μL of FITC-Tat peptide (1 mg/mL) was added to the mixture and stirred for 3 h under N_2_ protection at room temperature to obtain the final Tat-conjugated SiO_2_@Fe_3_O_4_ NPs (Tat-SiO_2_@Fe_3_O_4_ NPs) which were lyophilized for further observation after centrifugation and washing with water.

### Characterizations of Nanoparticles

The morphology of synthesized nanoparticles was characterized under a Philips JEM-2100HC transmission electron microscope (TEM) with an accelerating voltage of 150 kV. X-ray powder diffraction (XRD) patterns were recorded on an X’Pert-Pro diffractometer (PANalytical, Holland) in the 2q range from 20° to 80°. Element analysis was carried on a PHI-Quantum 2000 X-ray photoelectron spectroscopy (Physical Electronics, Inc, Japan). The hydrodynamic diameter and zeta potential of the as-synthesized NPs were measured on a Nano-ZS Zetasizer dynamic light scattering (DLS) detector (Malvern Instruments, UK), and the results were analyzed using the Malvern Zetasizer software assuming a log normal distribution. The magnetic properties of the products were characterized by vibrating sample magnetometry (VSM) in an applied magnetic field sweeping from −18 to 18 kOe. The elemental composition of the nanoparticles was characterized by Quantum 2000 X-ray photoelectron spectroscopy (XPS, PHI, US). Fourier transform infrared spectroscopy (FTIR) spectra of the samples were recorded on Nicolet-APTMSVATAR360 spectrometer in the range 4000–400 cm^−1^ using the KBr-disk method. The thermo-gravimetric analysis (TGA) was taken at a heating rate of 10 °C min^−1^ in a nitrogen atmosphere with a Pyris Diamond TGA thermal analyzer (PerkinElmer, Massachusetts, USA).

Additionally, iron content in nanoparticles or cell samples was determined by a colorimetric assay based on chromogenic reaction of Fe(III) with potassium thiocyanate. For a typical sample, 0.1 mL suspension was successively incubated with sodium hydroxide (0.1 mL, 5 mol/L) for 1 h at 80 °C, concentrated hydrochloric acid (0.1 mL, 12 mol/L) for 2 h at 55 °C, and excess ammonium persulfate (100 μg/mL) for 15 min at room temperature to remove SiO_2_ coating and oxidize Fe element in samples into Fe(III). After 10 min incubation of Fe(III) with potassium thiocyanate (0.1 mol/L), a red complex iron-thiocyanate form and the absorbance were detected using UV-vis spectrophotometer at the wavelength of 478 nm. Serial Fe(III) dilutions in the range 0–80 μg/mL were prepared to obtain a standard. Then, Fe content in sample is calculated as the absorbance from a standard curve. In the following experiments, amount of NPs was directly accounted as content.

### Construction and Characterization of BBB Models

To construct the BBB models, hCMEC cells were seeded on uncoated PET membrane of Transwell filter insides (1 μm pore size) in 500 μL medium at 1 × 10^5^ cells/well. Cells were cultured with the RPMI 1640 medium at 37 °C and in 5 % CO_2_, and the medium was changed every 2 days. After 7–10 days, a confluent hCMEC monolayer was generated. U251 cell monolayer was selected as the control.

For the characterization of the BBB model, the cell morphology of monolayer was initially observed by phase contrast optical microscopy. Immune-staining of tight junction-associated protein ZO-1 was performed to determine the BBB tight junction. Briefly, the cell monolayer on Transwell inserts was clipped and washed with PBS and then fixed in 4 % paraformalclehyde for 30 min and blocked with 1 % BSA for further 30 min. After washing with PBS, a monoclonal rabbit anti-ZO-1 antibody (diluted 1/50 in 1 % BSA in PBS, 300 μL) was added and incubated for 30 min. Next, TRITC conjugated mouse anti-rabbit IgG (diluted 1/50 in 1 % BSA in PBS, 300 μL) was added and incubated for 1 h. After washing the cells, the samples were stained with Hoechst (10 μg/mL, 300 μL). The tightness of the cellular barrier is further assessed by transendothelial electrical resistance (TEER) of monolayer. Only the monolayers with TEER over 150 Ω were used for studies.

BBB permeability was estimated by examining the transport of disodium salt (Na-F) and horseradish peroxidase (HRP) through monolayers for various periods. Fluorescence microcopy was used to examine the amount of Na-F. Permeability coefficient (*Pe*) values of Na-F through BBB mode can be determined by the equation $\frac{1}{P}=\frac{1}{Pt}-\frac{1}{Pf}$ where *Pt* and *Pf* correspond to the permeability coefficient values of Na-F through cell monolayer and control filter, respectively. HRP penetration of monolayers was examined as previously reported [31]. The experiment was repeated three times.

### Cytotoxicity

MTT assay was used to access in vitro cytotoxicity. The hCMEC cells were seeded into 96-well plate at 5 × 10^3^ cells/well and incubated for 24 h till the 70 % confluence. Then, Fe_3_O_4_@SiO_2_-Tat or Fe_3_O_4_@SiO_2_-Amino NPs with different concentrations (100, 200, 400, 600, and 800 μg/mL) were added to each well. The untreated hCMEC cells were used as control. After another 24 h incubating, a standard MTT was carried out according to the instructions.

### In Vitro Uptake Study Under Magnetic Field

The hCMEC cells were seeded in a 24-well plate at 2 × 10^5^ cells/well and cultured overnight. The cells were treated with 100 μg of Fe_3_O_4_@SiO_2_-Amino or Fe_3_O_4_@SiO_2_-Tat NPs with external magnetic field placed with a magnet on cell plate for varied periods (0, 0.5, and 2). After incubation for a further periods (2 or 12 h), the cells were washed with PBS, then lysed. Total protein concentration was determined using the BCA protein assay kit. Uptake amounts of NPs were determined through measuring the element Fe.

### BBB Transport Experiment

For quantifying BBB penetrating efficiency, the hCMEC monolayers with TEER value over 150 Ω cm^2^ were selected as the BBB model of hCMEC cells. Two hundred micrograms of Fe_3_O_4_@SiO_2_-Amino or Fe_3_O_4_@SiO_2_-Tat NPs was added into the apical chambers and incubated for 24 h. Then, each well was irradiated with external magnetic field for varied periods (0, 0.5, 2, and 8 h). RPMI 1640 medium was used as the blank control. After removing external magnetic field, the NPs were added and incubated for another 26 h. TEER of monolayer was detected in the interval of 1 h and a plot of the TEER—time curve was made. After washing with PBS, the hCMEC cells for each variable (NPs only, NPs + magnet and irradiation time) were lysed and transportation efficiency was assessed through determining the ratio of the element Fe in bottom chambers.

### Penetrating Through a hCMEC Monolayer and Further Location in U251 Cells

For construction of co-culture BBB models, hCMEC cells were seeded on Transwell insides at a density of 1 × 10^5^ cells and incubated for about 8 days to form a compact monolayer. Subsequently, U251 glioma cells were seed at the bottom of the Transwell at 1.5 × 10^5^ cells/well and cultured for 24 h till the 70 % confluence. Until the resistance of hCMEC monolayer was examined over 150 Ω cm^2^, 200 μg of FITC-labeled Fe_3_O_4_@SiO_2_-Amino and Fe_3_O_4_@SiO_2_-Tat NPs was added to the apical chambers of the BBB models. Subsequently, each well was irradiated for 2 h under external magnetic field. After incubation for another 24 h, U251 cells on the bottom chambers were washed in PBS and harvested. To determine the penetration ability of NPs, the cells for each variable FITC-labeled NPs (NPs only and NPs + magnet) were collected and the penetrating ability was evaluated though quantifying the cellular uptake of the U251 cells with a flow cytometer (Beckman Coulter, USA). Additionally, confocal laser scanning microscope (CLSM) is also used for observation the internalization and location of FITC-labeled NPs. The cells for each variable FITC-labeled NPs (NPs only and NPs + magnet) were collected and fixed with paraformaldehyde (4 % in PBS) for 30 min. Then, cells were treated with Hoechst 33258 (10 mg/mL) for 20 min to stain the nucleus. An inverted CLSM (FluoviewFV1000, Olympus, Japan) equipped with a Plan-Apochromat 60 × 0.7 NA lens was used to observe the samples.

### Statistical Analysis

Statistical analysis was conducted using a two-tailed unpaired Student’s *t* test. The results were presented as mean ± standard deviation.

## Results and Discussion

### Synthesis and Characterization of NPs

**Fig. 1:**
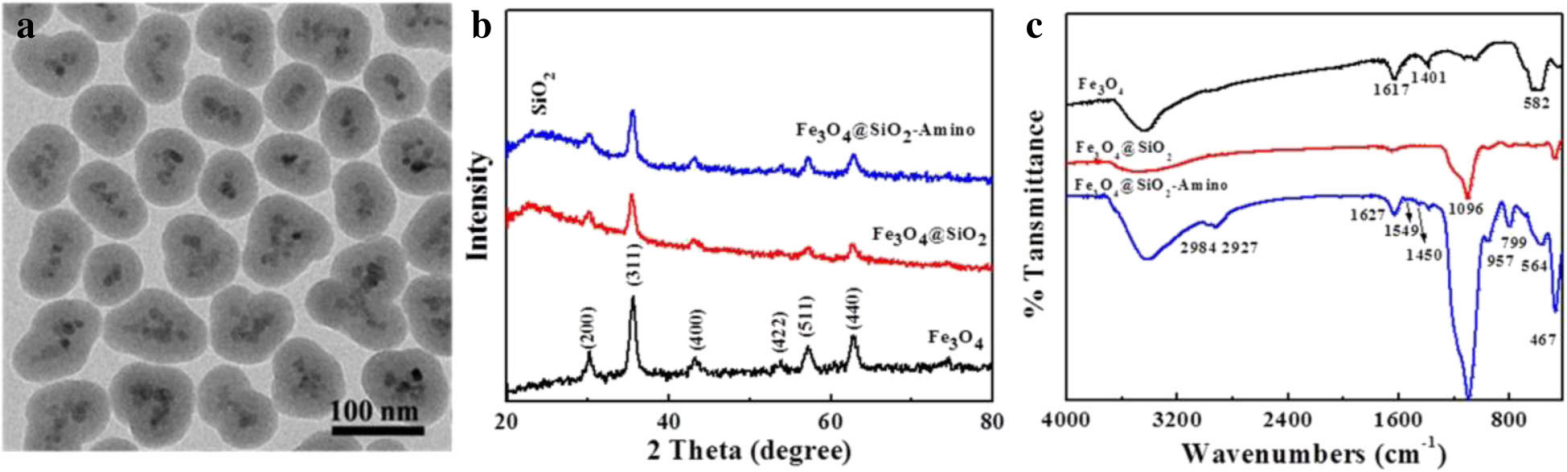
**a** TEM images of Fe_3_O_4_@SiO_2_ NPs; **b** the X-ray diffraction patterns; and **c** the FTIR spectra of Fe_3_O_4_, Fe_3_O_4_@SiO_2_, and Fe_3_O_4_@SiO_2_-Amino NPs

The strategy to prepare core-shell SiO_2_@Fe_3_O_4_-Tat NPs involves four steps, consisting of synthesis of Fe_3_O_4_ nanoparticles by co-precipitation, growth of a silica shell, surface amination, followed by conjugation of Tat peptide through EDC/NHS coupling reactions, as depicted in Scheme 1. Fe_3_O_4_ NPs were easily coated with a uniform SiO_2_ shell by the Stöber method [30]. TEM image in Fig. 1a revealed a spheroid and uniform morphology of the Fe_3_O_4_@SiO_2_ NP. An approximately 20 nm of black central portion of the probe is the magnetic nucleus with a larger electron density, and the surrounding gray portion of 4 nm thick is the amorphous SiO_2_ wrap. XRD patterns of the Fe_3_O_4_, Fe_3_O_4_@SiO_2_ NPs, and Fe_3_O_4_@SiO_2_-Amino NPs are shown in Fig. 1b. Six diffraction peaks at 2θ = 30.4, 35.7, 43.4, 53.7, 57.4, and 62.7 corresponded to the (220), (311), (400), (422), (511), and (440) planes of the inverse cubic spinel structure of Fe_3_O_4_, respectively (JCPDS card no. 09-0432). The presence of a broader peak at 2θ = 23 illustrated the amorphous silica coating on Fe_3_O_4_ NPs. No evident differences were observed for Fe_3_O_4_@SiO_2_ and Fe_3_O_4_@SiO_2_-Amino NPs, suggesting that amino modification of Fe_3_O_4_ NPs did not lead to any crystal phase change.

**Fig. 2:**
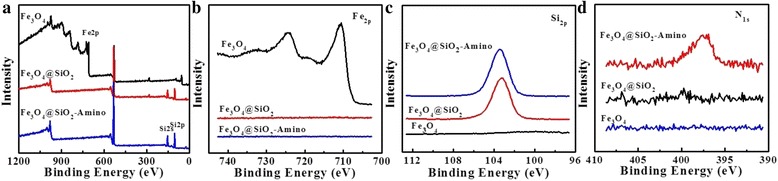
**a** XPS survey scan spectra; **b** Fe2p, **c** Si2p, and **d** N1s of Fe_3_O_4_, Fe_3_O_4_@SiO_2_, and Fe_3_O_4_@SiO_2_-Amino NPs

Successive introduction of amino group was confirmed by FTIR analyses. FTIR spectra of the Fe_3_O_4_, Fe_3_O_4_@SiO_2_, and Fe_3_O_4_@SiO_2_-Amino NPs were examined and shown in Fig. 1c. The characteristic bands at 582 cm^−1^ corresponded to the vibration of the Fe–O bonds. The bands at 1617 and 1401 cm^−1^ were assigned to the stretching vibrations of carboxyl salt, suggesting the presence of coordinative effect in the Fe (III)-carboxylate group [32]. The characteristic absorption bands at 1096, 799, and 467 cm^−1^ for Si–O–Si group confirmed that the Fe_3_O_4_ NPs were encapsulated by a layer of silica. The IR spectrum of Fe_3_O_4_@SiO_2_-Amino NPs showed that the weak bands at 1549 cm^−1^ belonged to the bending vibration of amine group and two broad bands at 2984 and 2927 cm^−1^ corresponded to C–H stretching vibration. The presence of a band at 798 cm^−1^ may be attributed to the Si–C stretching vibrations, indicating the conjugation of amide groups with the silica framework via the APTES hydrolysis [33]. Moreover, the XPS was used to explore the elemental compositions of functional Fe_3_O_4_ NPs. As shown in Fig. 2, the Fe 2p and Si 2p XPS patterns appeared on spectrum of bare Fe_3_O_4_ and Fe_3_O_4_@SiO_2_ NPs, respectively, which can be assigned to SiO_2_ coating. The N1s binding energy peak at 397.5 eV only was presented in XPS spectrum of Fe_3_O_4_@SiO_2_-Amino NPs, suggesting successful APTES conjugation. It is also found that a peak at 284.9 eV on total scan spectra can be assigned to C–C bonds of citrate ions [33].

**Fig. 3:**
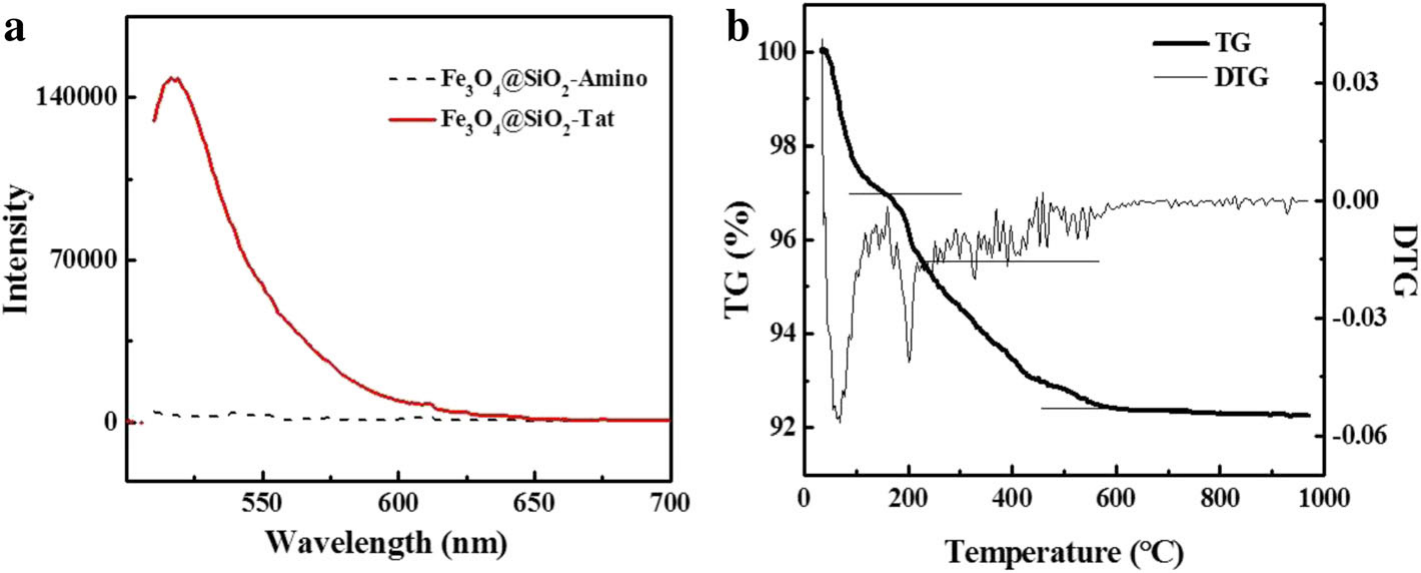
**a** The fluorescence spectra of Fe_3_O_4_@SiO_2_-Amino and Fe_3_O_4_@SiO_2_-Tat NPs. **b** The TG and DTG curves of Fe_3_O_4_@SiO_2_-Tat NPs

Peptide Tat was covalently immobilized onto activated magnetic nanoparticles with EDC/NHS as shown in Scheme 1. FITC-labeled Tat peptide was used in the synthesis process to confirm the conjugation of Tat peptide. An obvious absorbance at 520 nm in the fluorescence spectrum of Fe_3_O_4_@SiO_2_-Tat NPs indicated the successfully conjugation of Tat peptide on the surface (Fig. 3a). TGA was used to quantitatively characterize the modification efficiency of Tat peptide onto the surface of Fe_3_O_4_@SiO_2_ NPs. TGA curves of Fe_3_O_4_@SiO_2_-Tat NPs in Fig. 3b showed three transitions during the temperature of (i) 25–100 °C, (ii) 100–230 °C, and (iii) 230–550 °C. The first weight loss of 2.51 % was associated to the surface water removal, the second one (1.73 %) indicated the decomposition of Tat peptide, while the last section (3.30 %) corresponded to the thermal decomposition of grafted APTES. No further weight loss occurred with the increase of temperature and total organic matters of Fe_3_O_4_@SiO_2_-Tat NP weighted for 5.03 %.

Surface modification changes the particle size and surface charge but also influences the uptake ability and pathway of NPs into the cells. As shown in Table 1, hydrodynamic size of Fe_3_O_4_, Fe_3_O_4_@SiO_2_, and Fe_3_O_4_@SiO_2_-Amino and Fe_3_O_4_@SiO_2_-Tat NPs (23.7, −72.8, 76.4, and 87.6 nm, respectively) gradually increased along with the functionalization procedure. The larger hydrodynamic sizes than TEM values may be due that DLS measures large aggregates in aqueous solution while TEM just measures single Fe_3_O_4_ NP. On the other hand, Fe_3_O_4_@SiO_2_ NPs showed less negative charges (−15.4 mV) than that of bare Fe_3_O_4_ NPs (−23.7 mV) due to the shielding effect of silica coating on citrate ions. And, the remaining negativity might be attributed to the existence of a large amount of silanol groups on surface. After surface amination of APTES, the zeta potential of Fe_3_O_4_@SiO_2_-Amino NPs become toward positive position (24.9 mV) due to the introduction of amino. After further conjugating with cationic peptide Tat, the averaged zeta potential was 42.1 mV, which was beneficial to efficient entrapment of negative cell delivery.

**Table 1:** Zeta potential and particle size

Nanoparticles	Zeta potential (mV)	Size (nm)
Fe_3_O_4_	−23.7 ± 1.87	23.7 ± 2.06
Fe_3_O_4_@SiO_2_	−15.4 ± 1.05	72.8 ± 2.54
Amino-Fe_3_O_4_@SiO_2_	24.9 ± 2.05	76.4 ± 1.27
Tat-Fe_3_O_4_@SiO_2_	42.1 ± 2.25	87.6 ± 3.23

**Fig. 4:**
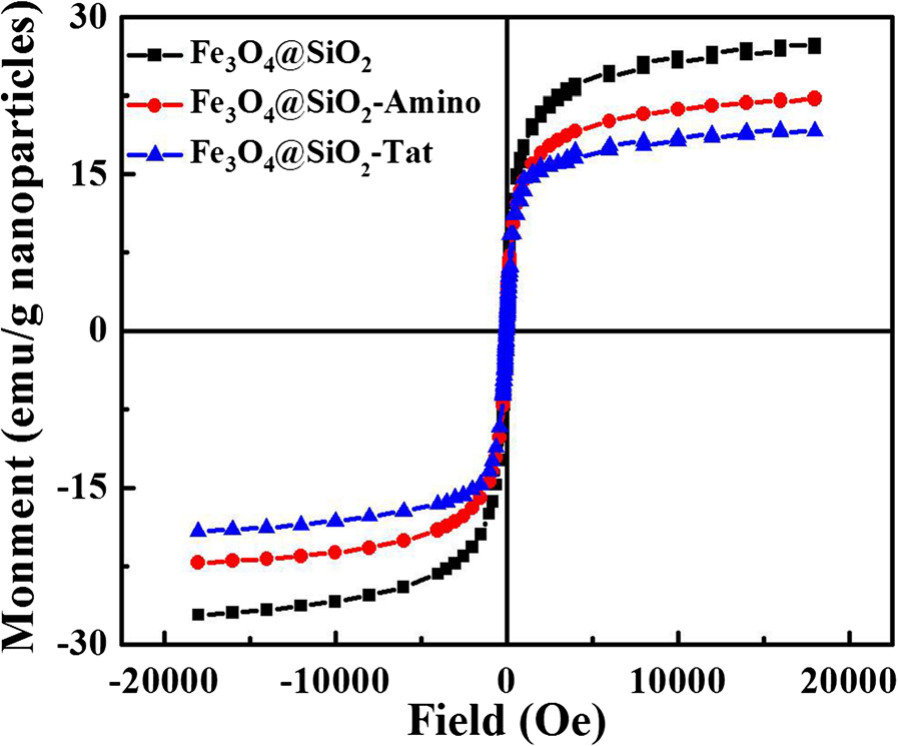
Normalized magnetization curves of Fe_3_O_4_@SiO_2_, Fe_3_O_4_@SiO_2_-Amino, and Fe_3_O_4_@SiO_2_-Tat NPs

Finally, the magnetic properties of the three types of modified MNPs mentioned above were measured by VSM at room temperature. No remnant magnetization and hysteresis were shown in Fig. 4, suggesting their superparamagnetic property. The saturation magnetization (Ms) of Fe_3_O_4_@SiO_2_, Fe_3_O_4_@SiO_2_-Amino, and Fe_3_O_4_@SiO_2_-Tat NPs were 27.4, 22.1, and 19.21 emu g^−1^, respectively. The gradual decrease in Ms is due to an increase in the thickness of Fe_3_O_4_ coating along with the functionalization procedure. And, magnetic response of Fe_3_O_4_@SiO_2_-Tat NPs was large enough to quickly separate particles from solution by the magnet (Fig. 4, inset). This property could endow Fe_3_O_4_@SiO_2_-Tat NPs with magnetically mediated tumor targeting.

### Characterization of the BBB Model

The confluent hCMEC monolayers were used to prepare in vitro BBB model which can be assessed through some indicators such as morphological, tight junction protein staining, TEER value measurements, and permeability studies.

**Fig. 5:**
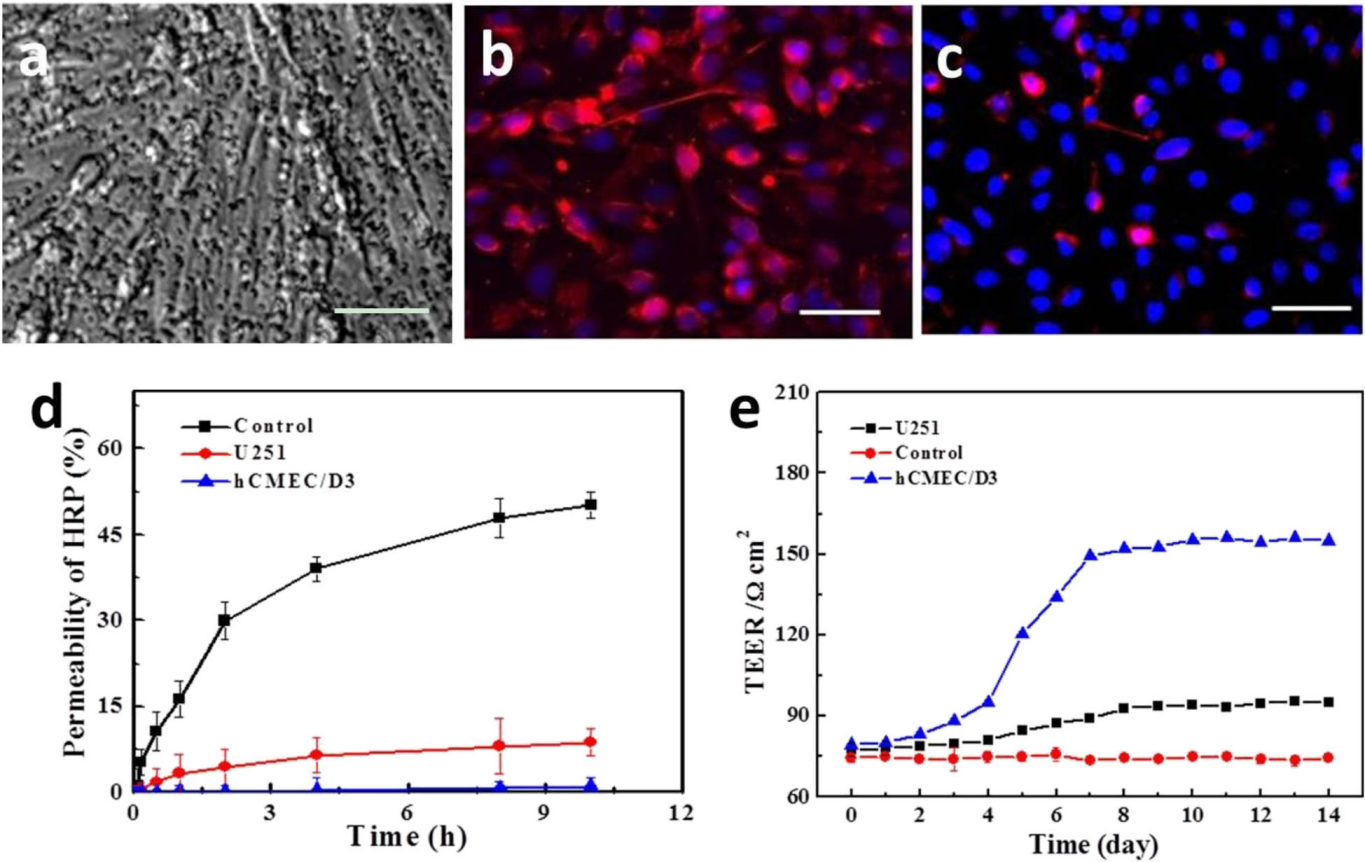
**a** Images of BBB model at the eighth day; immune-staining of tight joint—ZO-1 for hCMEC monolayer (**b**) and U251 control (**c**) at the eighth day; **d** The permeability of HRP. **e** TEER measurement on the hCMEC monolayer, U251 monolayer, and PET membrane control. *Bar*: 50 μm

As shown in Fig. 5a, on day 8 of culture, the confluent hCMEC cells form a monolayer without aperture as it was seen on a phase contrast optical microscopy. The high expression of zonula occludens was the characteristic of the confluent brain endothelial cell monolayers. An immune-staining of the endothelial specific tight junction protein ZO1 (Fig. 5b, c) revealed an increasing amount of punctuated fluorescence at intracellular regions along with the growth of cells, which confirmed that the tight junctions had been correctly assembled.

BBB integrity was evaluated using Na-F and HRP exogenous tracers [34]. PS values of Na-F were calculated to be 1.66 and 23.84 × 10^−3^ cm/min for hCMEC and U251 cells, respectively (data not shown), in accordance with previously published data [35]. The permeability of HRP on BBB is only 1.05 % within 10 h, while up to 50 % of HRP had transfer across monolayer of U251 cells and the value increased with longing time (Fig. 5d). The low permeability of hCMEC monolayer meant a strong restriction for small hydrophilic molecules and macromolecule tracers, illustrating the tightening of BBB model.

The TEER is commonly used to assess the integrity of brain endothelial cell monolayer. Previous studies indicated that over 120 Ω × cm^2^ of TEER value can account for the in vitro BBB integrity [36]. In our model, a higher TEER value above 150 Ω × cm^2^ was monitored on 8 days and it will not change evidently in the next week (Fig. 5e), suggesting the confluent monolayer could be used as an in vitro model for exploring the NPs transport.

### In Vitro Cytotoxicity

**Fig. 6:**
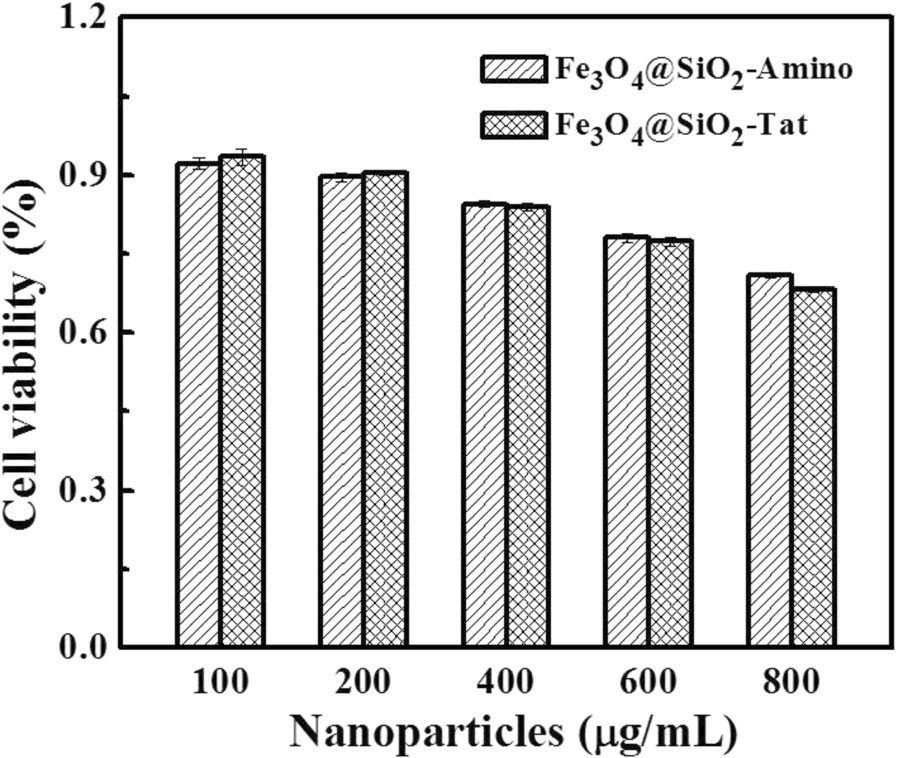
Normalized dose–response for cell viability of Fe_3_O_4_@SiO_2_-Amino and Fe_3_O_4_@SiO_2_-Tat NPs by MTT assay

The cytotoxicity of NPs remains a matter of concern in their application as drug carrier. MTT assay was commonly used to determine the cytotoxicity in a concentration-dependent manner. As shown in Fig. 6, Fe_3_O_4_@SiO_2_-Tat and Fe3O_4_@SiO_2_-Amino NPs maintained above 70 % of the cell viability in the tested concentrations, and the cytotoxicity appeared in dose-dependent manner. Besides, Tat-conjugated NPs showed a slight decrease of cell viability relative to Fe_3_O_4_@SiO_2_-Amino NPs, due to a reduced membrane trans-locating activity from Tat [37]. During the tested concentrations below 800 mg/mL, the NPs were non-cytotoxic.

### In Vitro Uptake Study Under Magnetic Field

To evaluate the possibility of NPs across the BBB, we first determined whether Fe_3_O_4_@SiO_2_-Tat NPs can be internalized into brain endothelial cells in the present or absent of magnetic field. Flow cytometry experiments were used to quantity the internalization of the Tat-conjugated and non-conjugated NPs inside the cells.

**Fig. 7:**
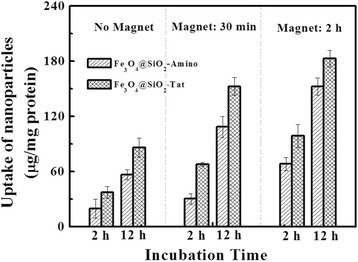
The effect of external magnetic field (permanent magnet) on the uptake of magnetic nanoparticles by hCMEC cells

The different cell uptake percentage has been displayed in Fig. 7, as a result of the differently grafted group and explosion time to magnetic field. When a magnetic force was exerted for 0.5 or 2 h, cell internalization had been shown to increase by 1.8/2.6 times for Fe_3_O_4_@SiO_2_-Tat NPs and 1.5/3.5 times for Fe_3_O_4_@SiO_2_-Amino NPs, respectively. The result suggested a magnet aid endocytosis process. Moreover, the cellular uptake of both NPs by hCMEC cells was increased by two to three times with incubation time prolonging from 2 to 12 h, indicating a time-dependent cellular internalization. Besides, it has also been found that conjugation with peptide Tat could enhance by 1.2–2.2 times cellular uptake on varied explosion time (0–2 h) relative to Fe_3_O_4_@SiO_2_-Amino NPs, suggesting that Tat peptide could increase internalization efficiency of NPs into brain capillary endothelial cells by Tat-mediated membrane destabilization.

### Passage of NPs Through BBB

A major problem of targeting the brain lies in the poor BBB penetration. Therefore, the transport of NPs over the BBB is a critical issue. In our experiment, NPs were added in the apical chamber of in vitro BBB model. If transcytosis of NPs occurs, they will transfer across the filters of Transwell and then be found in the bottom chamber. By analyzing the ratio of trans-cellular nanoparticles to adding amount, we can rank their transport efficiencies.

**Fig. 8:**
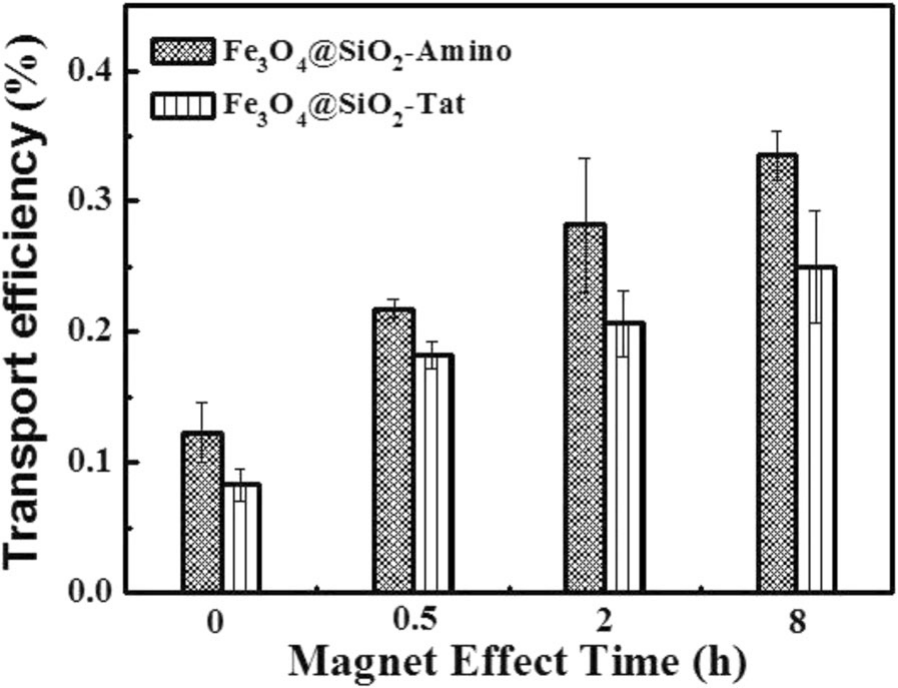
Transport efficiency of Fe_3_O_4_@SiO_2_-Tat and Fe_3_O_4_@SiO_2_-Amino NPs

It is indicated from Fig. 8 that both NPs are able to effectively cross over BBB in the absence of magnetic field. And, transport efficiencies were increased with exposure time of magnetic field longing. Fe_3_O_4_@SiO_2_-Amino increased 17 % passage ratio after applying a magnetic force for 8 h, while Fe_3_O_4_@SiO_2_-Tat NPs was enhanced 1.8-, 2.3-, and 2.8-fold at the exposure periods of 0.5, 2, and 8 h, respectively, compared with that without external magnetic force. This result indicated a magnetic field-increased permeability of NPs across hCMEC monolayer. Under the same magnetic conditions, Tat-conjugated Fe_3_O_4_@SiO_2_-Amino induced greater passage ratio than non-conjugated ones. The increase could result from an enhanced recognition and affinity to the cells which sequentially triggered receptor-mediated endocytosis and increased accumulation of nanoparticles in cells and the transportation [38]. It also was reported that some PIONs covered by biomolecules accumulated at a higher concentration in the tumors when they were subjected to an external magnetic field [27]. Hence, our prepared Fe_3_O_4_@SiO_2_-Tat NPs have the potential to be magnetically mediated brain-targeting carrier.

**Fig. 9:**
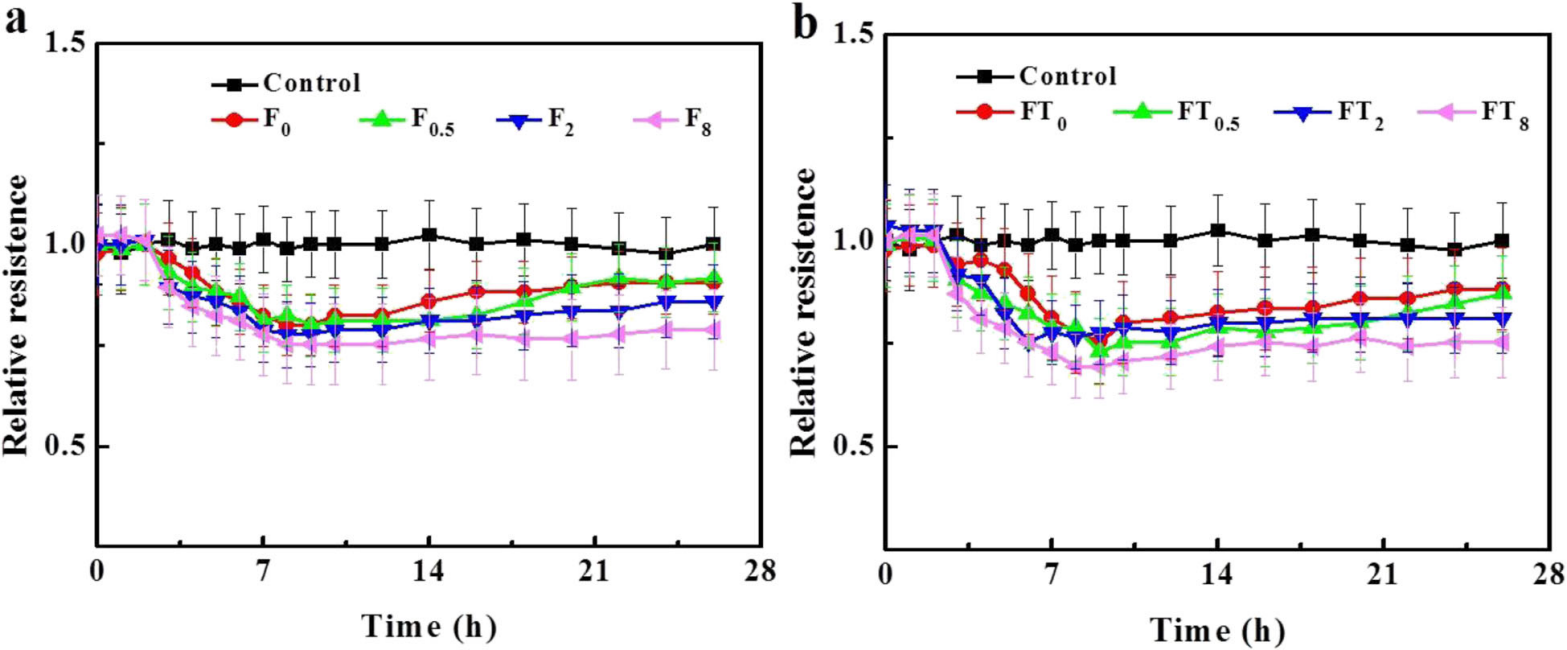
Normalized TEER values of BBB model recorded after the introduction of nanoparticles with magnetic explosion periods. **a** Fe_3_O_4_@SiO_2_-Amino NPs (F). **b** Fe_3_O_4_@SiO_2_-Tat NPs (FT)

The effect of NPs across BBB was further evaluated by monitoring the changes of the TEER before and after the existence of NPs under the external magnetic field. As shown in Fig. 9, the TEER values exhibited a similar variation tendency, declining at 2 h incubation, and reaching to the minimum at 8 h, then gradually recovering and basically reaching more than 75 % of the initial value at 26 h. This observation in good agreement with the previous report [39] may be due to the fluctuations of cell monolayer when introduction of extracellular matters. Besides, the reduction of TEER values increased with exposure time longing, and it is inversely to recovery rate. NPs passage and accumulation did not harm the integrity of in vitro BBB model.

### Uptake and Location in U251

**Fig. 10:**
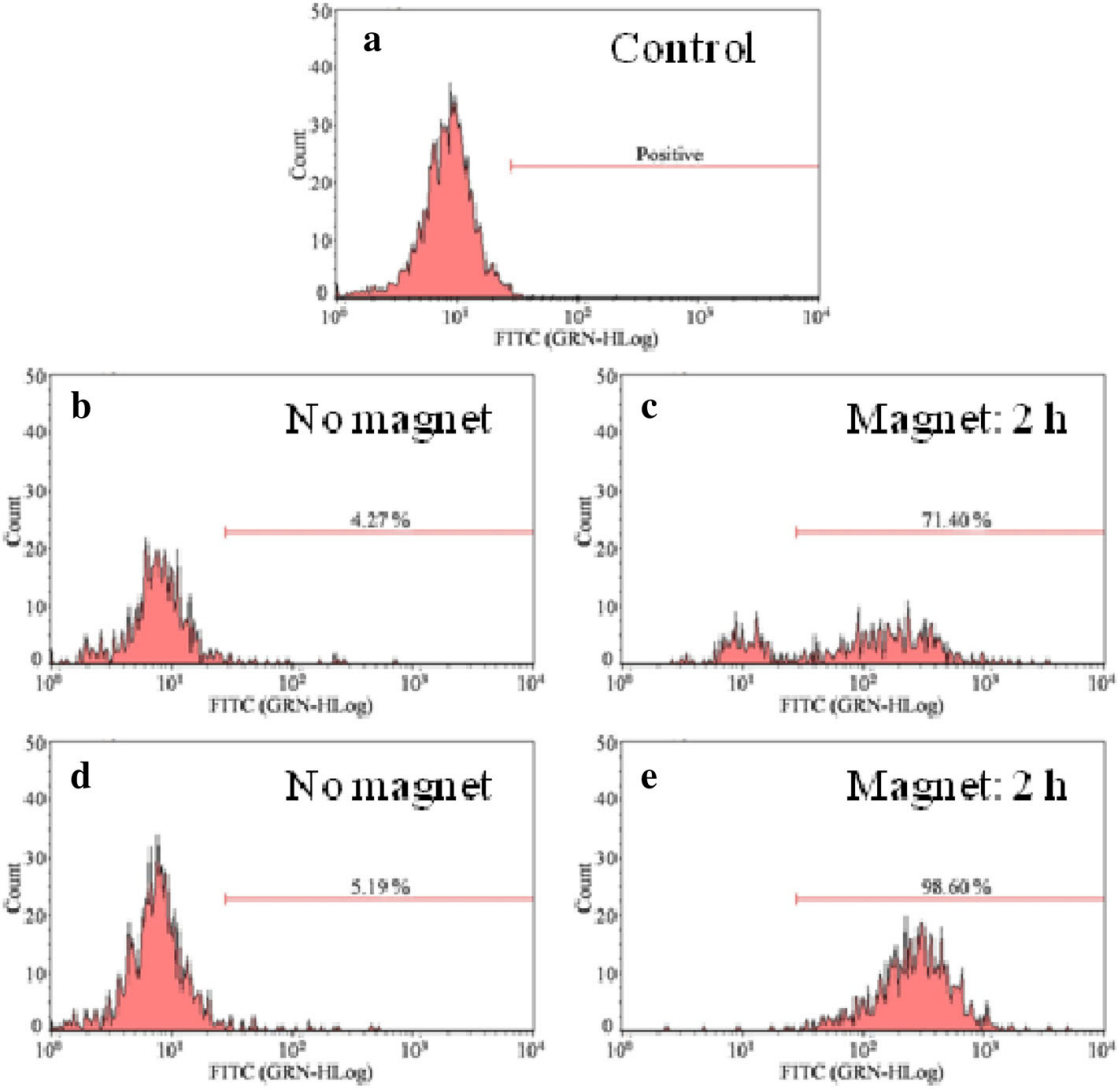
Flow cytometry of U251 cells containing NPs. **a** Control group; Fe_3_O_4_@SiO_2_-Amino NPs, **b** in the absence of magnetic field, and **c** under magnetic explosion for 2 h; Fe_3_O_4_@SiO_2_-Tat NPs, **d** in the absence of magnetic field and **e** under magnetic explosion for 2 h. Higher intensity along the *x* axis indicates more FACS delivery, and percentage of cells with internalized NPs is indicated

For an efficient brain-targeting therapy, the internalization and accumulation of carriers in brain cells are also important. To mimic a more realistic in vivo situation, U251/hCMEC co-culture BBB model was built and used to investigate uptake and location of nanoparticles in U251 cells in the bottom chamber. As shown in Fig. 10, Fe_3_O_4_@SiO_2_-Tat and Fe_3_O_4_@SiO_2_-Amino NPs, respectively, increased 17.4- and 19.0-fold uptake by U251 cells after 2 h explosion of magnetic field, compared with no-magnet groups of 4.27 and 5.19 %. The result suggested the application of external magnetic field greatly facilitated NPs internalization into the cells within the brain. Moreover, the internalization of Fe_3_O_4_@SiO_2_-Tat was higher than that of Fe_3_O_4_@SiO_2_-amino NPs whether or not to apply a magnetic field, further reflecting trans-membrane effect of Tat peptide.

**Fig. 11:**
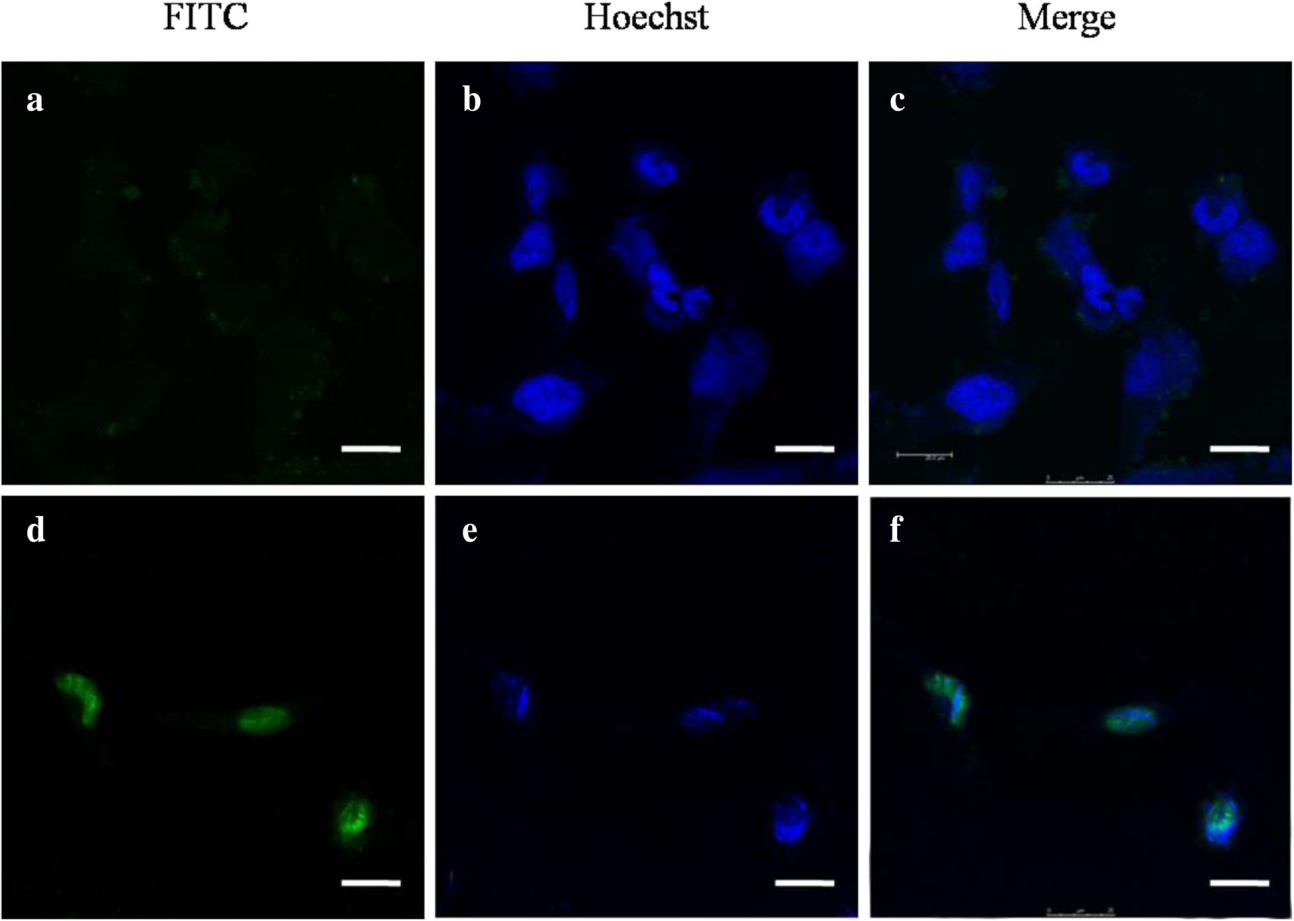
The localization of nanoparticles in U251 cells. **a**–**c** Fe_3_O_4_@SiO_2_-Amino NPs. **d**–**f** Fe_3_O_4_@SiO_2_-Tat NPs

For detecting the ultimate fate of NPs after passage over BBB, FITC-labeled NPs was used as an optical probe, and their intracellular localization in U251 cells were analyze by CLSM after 2 h explosion of magnetic field. As shown in Fig. 11, much more green dots of Fe_3_O_4_@SiO_2_-Tat distributed within cytoplasm compared with non-conjugated ones, consistently in FCM results. Moreover, the merged yellow fluorescent dots demonstrated that almost of Tat-conjugated Fe_3_O_4_@SiO_2_-Amino NPs accumulated in nucleus zone, indicating that Fe_3_O_4_@SiO_2_-Tat NPs can enter into cell nucleus. In contrast, no green dots of Fe_3_O_4_@SiO_2_-Amino NPs were detected in the blue nucleus district. It has been reported that partly Tat-grafted nanoparticles can be cross over nuclear membrane [40, 41]. Our findings may suggest that a synergy effect of magnetic force and Tat peptide can enhance cell internalization and nucleus targeting.

## Conclusions

To sum up, Tat-conjugated SiO_2_@Fe_3_O_4_ NPs exhibited suitable magnetism and good biocompatibility. The functionalization with Tat peptide facilitated particles to pass through in vitro BBB and enter into the nucleus of U251 glioma cells co-cultured at the bottom chamber. Moreover, the synergistic effects of Tat and magnetic field could promote the cell uptake of hCMEC cells and entry to nuclear of U251, hence efficiently enhancing permeability across BBB and subsequent accumulation in glioma cells. It also found that Fe_3_O_4_@SiO_2_-Tat NPs can be transported through the in vitro BBB via a trans-cellular trafficking mechanism and magnetically mediated dragging. Therefore, SiO_2_@Fe_3_O_4_-Tat NPs could be exploited as a potential brain-targeting carrier for diagnosis and treatment of the brain disease.

## References

[1] Obermeier B, Daneman R, Ransohoff RM (2013). Development, maintenance and disruption of the blood-brain barrier. Nat Med.

[2] Jiang X (2013). Brain drug delivery systems. Pharm Res-Dordr.

[3] Clark AJ, Davis ME (2015). Increased brain uptake of targeted nanoparticles by adding an acid-cleavable linkage between transferrin and the nanoparticle core. Proc Natl Acad Sci U S A.

[4] Nomura T, Nishimura Y, Gotoh H, Ono K (2016). Rapid and efficient gene delivery into the adult mouse brain via focal electroporation. Sci Rep.

[5] Jackson S, Anders NM, Mangraviti A, Wanjiku TM, Sankey EW, Liu A, Brem H, Tyler B, Rudek MA, Grossman SA (2016). The effect of regadenoson-induced transient disruption of the blood–brain barrier on temozolomide delivery to normal rat brain. J Neurooncol.

[6] Mason WP (2015). Blood-brain barrier-associated efflux transporters: a significant but underappreciated obstacle to drug development in glioblastoma. Neuro Oncol.

[7] Trippier PC (2016). Properties to optimize small molecule blood-brain barrier penetration. Curr Med Chem.

[8] Lajoie JM, Shusta EV (2015). Targeting receptor-mediated transport for delivery of biologics across the blood-brain barrier. Annu Rev Pharmacol.

[9] Mo J, He L, Ma B, Chen T (2016). Tailoring particle size of mesoporous silica nanosystem to antagonize glioblastoma and overcome blood-brain barrier. ACS Appl Mater Inter.

[10] Mangraviti A, Gullotti D, Tyler B, Brem H (2016). Nanobiotechnology-based delivery strategies: new frontiers in brain tumor targeted therapies. J Control Release.

[11] Zhao X, Wang J, Tao S, Ye T, Kong X, Ren L (2016). In vivo bio-distribution and efficient tumor targeting of gelatin/silica nanoparticles for gene delivery. Nanoscale Res Lett.

[12] Estelrich J, Escribano E, Queralt J, Busquets MA (2015). Iron Oxide Nanoparticles for Magnetically-Guided and Magnetically-Responsive Drug Delivery. Int J Mol Sci.

[13] Orlando A, Colombo M, Prosperi D, Gregori M, Panariti A, Rivolta I, Masserini M, Cazzaniga E (2015). Iron oxide nanoparticles surface coating and cell uptake affect biocompatibility and inflammatory responses of endothelial cells and macrophages. J Nanopart Res.

[14] Huang Y, Zhang B, Xie S, Yang B, Xu Q, Tan J (2016). Superparamagnetic iron oxide nanoparticles modified with Tween 80 pass through the intact blood-brain barrier in rats under magnetic field. ACS Appl Mater Inter.

[15] Dan M, Bae Y, Pittman TA, Yokel RA (2015). Alternating magnetic field-induced hyperthermia increases iron oxide nanoparticle cell association/uptake and flux in blood–brain barrier models. Pharm Res-Dordr.

[16] Gräfe C, Weidner A, Lühe MV, Bergemann C, Schacher FH, Clement JH, Dutz S (2016). Intentional formation of a protein corona on nanoparticles: serum concentration affects protein corona mass, surface charge, and nanoparticle–cell interaction. Int J Biochem Cell Biol.

[17] Kiliç G, Costa C, Fernández-Bertólez N, Pásaro E, Teixeira JP, Laffon B, Valdiglesias V (2016). In vitro toxicity evaluation of silica-coated iron oxide nanoparticles in human SHSY5Y neuronal cells. Toxicol Res-UK.

[18] Tang Y, Zhang C, Wang J, Lin X, Zhang L, Yang Y, Wang Y, Zhang Z, Bulte J, Yang GY (2015). MRI/SPECT/fluorescent tri-modal probe for evaluating the homing and therapeutic efficacy of transplanted mesenchymal stem cells in a rat ischemic stroke model. Adv Funct Mater.

[19] Arami H, Khandhar A, Liggitt D, Krishnan KM (2015). In vivo delivery, pharmacokinetics, biodistribution and toxicity of iron oxide nanoparticles. Chem Soc Rev.

[20] Richard S, Boucher M, Herbet A, Lalatonne Y, Mériaux S, Boquet D, Motte L (2015). Endothelin B receptors targeted by iron oxide nanoparticles functionalized with a specific antibody: toward immunoimaging of brain tumors. J Mater Chem B.

[21] Komin A, Russell LM, Hristova KA, Searson PC (2016) Peptide-based strategies for enhanced cell uptake, transcellular transport, and circulation: mechanisms and challenges. Adv Drug Deliv Rev, doi: 10.1016/j.addr.2016.06.002 10.1016/j.addr.2016.06.002 27313077

[22] Zhang D, Wang J, Xu D (2016). Cell-penetrating peptides as noninvasive transmembrane vectors for the development of novel multifunctional drug-delivery systems. J Control Release.

[23] Cai Q, Wang L, Deng G, Liu J, Chen Q, Chen Z (2016). Systemic delivery to central nervous system by engineered PLGA nanoparticles. Am J Transl Res.

[24] Goswami D, Vitorino HA, Alta RY, Silvestre DM, Nomura CS, Machini MT, Espósito BP (2015). Deferasirox-TAT(47–57) peptide conjugate as a water soluble, bifunctional iron chelator with potential use in neuromedicine. BioMetals.

[25] Xu R, Feng X, Xie X, Zhang J, Wu D, Xu L (2012). HIV-1 Tat protein increases the permeability of brain endothelial cells by both inhibiting occludin expression and cleaving occludin via matrix metalloproteinase-9. Brain Res.

[26] Kanazawa T, Morisaki K, Suzuki S, Takashima Y (2014). Prolongation of life in rats with malignant glioma by intranasal siRNA/drug codelivery to the brain with cell-penetrating peptide-modified micelles. Mol Pharm.

[27] Thomsen LB, Linemann T, Pondman KM, Lichota J, Kim KS, Pieters RJ, Visser GM, Moos T (2013). Uptake and transport of superparamagnetic iron oxide nanoparticles through human brain capillary endothelial cells. ACS Chem Neurosci.

[28] Bicker J, Alves G, Fortuna A, Falcão A (2014). Blood–brain barrier models and their relevance for a successful development of CNS drug delivery systems: a review. Eur J Pharm Biopharm.

[29] Jitianu A, Raileanu M, Crisan M, Predoi D, Jitianu M, Stanciu L, Zaharescu M (2006). Fe3O4–SiO2 nanocomposites obtained via alkoxide and colloidal route. J Sol-Gel Sci Technol.

[30] Graf C, van Blaaderen A, Imhof A (2003). A general method to coat colloidal particles with silica. Langmuir.

[31] Xie Y, Ye L, Zhang X, Cui W, Lou J, Nagai T, Hou X (2005). Transport of nerve growth factor encapsulated into liposomes across the blood–brain barrier: In vitro and in vivo studies. J Control Release.

[32] Shen LH, Bao JF, Wang D, Wang YX, Chen ZW, Ren L, Zhou X, Ke X, Chen M, Yang AQ (2013). One-step synthesis of monodisperse, water-soluble ultra-small Fe3O4 nanoparticles for potential bio-application. Nanoscale.

[33] Viswanathan V, Murali G, Gandhi S, Kumaraswamy P, Sethuraman S, Krishnan UM (2014). Development of thioflavin-modified mesoporous silica framework for amyloid fishing. Micropor Mesopor Mat.

[34] Kaya M, Ahishali B, Turksen K (2011). Assessment of permeability in barrier type of endothelium in brain using tracers: Evans blue, sodium fluorescein, and horseradish peroxidase. Permeability barrier: methods and protocols.

[35] Nakagawa S, Deli MA, Kawaguchi H, Shimizudani T, Shimono T, Kittel A, Tanaka K, Niwa M (2009). A new blood–brain barrier model using primary rat brain endothelial cells, pericytes and astrocytes. Neurochem Int.

[36] Kang T, Jiang M, Jiang D, Feng X, Yao J, Song Q, Chen H, Gao X, Chen J (2015). Enhancing glioblastoma-specific penetration by functionalization of nanoparticles with an iron-mimic peptide targeting transferrin/transferrin receptor complex. Mol Pharm.

[37] Vivès E, Brodin P, Lebleu B (1997). A truncated HIV-1 Tat protein basic domain rapidly translocates through the plasma membrane and accumulates in the cell nucleus. J Biol Chem.

[38] Liu L, Guo K, Lu J, Venkatraman SS, Luo D, Ng KC, Ling EA, Moochhala S, Yang YY (2008). Biologically active core/shell nanoparticles self-assembled from cholesterol-terminated PEG–TAT for drug delivery across the blood–brain barrier. Biomaterials.

[39] Varga N, Csapó E, Majláth Z, Ilisz I, Krizbai IA, Wilhelm I, Knapp L, Toldi J, Vécsei L, Dékány I (2016). Targeting of the kynurenic acid across the blood–brain barrier by core-shell nanoparticles. Eur J Pharm Sci.

[40] Tammam SN, Azzazy HME, Lamprecht A (2016). How successful is nuclear targeting by nanocarriers?. J Control Release.

[41] Peng LH, Niu J, Zhang CZ, Yu W, Wu JH, Shan YH, Wang XR, Shen YQ, Mao ZW, Liang WQ, Gao JQ (2014). TAT conjugated cationic noble metal nanoparticles for gene delivery to epidermal stem cells. Biomaterials.

